# Caesarean delivery-related blood transfusion: correlates in a tertiary hospital in Southwest Nigeria

**DOI:** 10.1186/s12884-017-1643-7

**Published:** 2018-01-10

**Authors:** Fatimat M. Akinlusi, Kabiru A. Rabiu, Idayat A. Durojaiye, Adeniyi A. Adewunmi, Tawaqualit A. Ottun, Yusuf A. Oshodi

**Affiliations:** Department of Obstetrics and Gynaecology, Lagos State University College of Medicine/Lagos State University Teaching Hospital, Ikeja, Lagos, Nigeria

**Keywords:** Blood transfusion, Caesarean delivery, Risk factors, Case-control study, Blood loss

## Abstract

**Background:**

Caesarean delivery carries a risk of major intra-operative blood loss and its performance is often delayed by non-availability of blood and blood products. Unnecessary cross-matching and reservation of blood lead to apparent scarcity in centres with limited supply. This study set out to identify the risk factors for blood transfusion in women who underwent caesarean delivery at a tertiary obstetric unit with a view to ensuring efficient blood utilization.

**Methods:**

A prospective cohort analysis of 906 women who had caesarean deliveries at the Lagos State University Teaching Hospital, Nigeria between January and December, 2011. A comparison was made between 188 women who underwent blood transfusion and 718 who did not. Data were obtained on a daily basis by investigators from patients, clinical notes and referral letters using structured pre-tested data collecting form. Socio-demographic characteristics; antenatal, perioperative and intraoperative details; blood loss; transfusion; and puerperal observations were recorded. EPI-Info statistical software version 3.5.3 was used for multivariable analysis to determine independent risk factors for blood transfusion.

**Results:**

Of the 2134 deliveries during the study period, 906 (42.5%) had caesarean deliveries and of which 188 (20.8%) were transfused. The modal unit of blood transfused was 3 pints (41.3%). The most common indication for caesarean section was cephalo-pelvic disproportion (25.7%).The independent risk factors for blood transfusion at caesarean section were second stage Caesarean Section (aOR = 76.14, 95% CI = 1.25–4622.06, *p* = 0.04), placenta previa (aOR = 32.57, 95% CI = 2.22–476.26, *p* = 0.01), placental abruption (aOR = 25.35, 95% CI = 3.06–211.02, *p* < 0.001), pre-operative anaemia (aOR = 12.15, 95% = CI 4.02–36.71, p < 0.001), prolonged operation time (aOR = 10.72 95% CI = 1.37–36.02, p < 0.001), co-morbidities like previous uterine scar (aOR = 7.02, 95% CI = 1.37–36.02, *p* = 0.02) and hypertensive disorders in pregnancy (aOR = 5.19, 95% CI = 1.84–14.68, p < 0.001). Obesity reduced the risk for blood transfusion (aOR = 0.24, 95% CI = 0.09–0.61, *p* = 0.0024).

**Conclusion:**

The overall risk of blood transfusion in cesarean delivery is high. Paturients with the second stage Caesarean section, placenta previa, abruptio placentae and preoperative maternal anaemia have an increased risk of blood transfusion. Hence, adequate peri-operative preparations for blood transfusion are essential in these situations. Optimizing maternal hemoglobin concentration during antenatal period may reduce the incidence of caesarean-associated blood transfusion.

## Background

Research has consistently identified haemorrhage as a major cause of direct maternal death, maternal near miss and maternal morbidity. The majority of these deaths occur from postpartum hemorrhage often in association with caesarean section [1, 2], a procedure that carries a risk of major intra-operative blood loss [3]. This has made caesarean section a common indication for blood transfusion in obstetric practice. Its performance, however, is often delayed by non-availability of blood [1, 4].

Blood transfusion remains a life-saving intervention despite its attendant risks. Limited blood supplies due to poor donor response; anaemia; donor blood wastage from viral contamination [5]; and rising costs continue to hinder availability, timely provision and utilization of blood and blood products. With limited units of donor blood and blood-banking services, judicious utilization is required to achieve the overall goal of blood transfusion.

In our centre, it is common practice to cross-match two or more units of blood for patients undergoing caesarean section. This blood is reserved and unavailable to other users, a practice which is commonplace in many centres in this sub-region [6].

Unnecessary cross-matching and reservation of blood not only incur additional cost but also result in apparent blood scarcity in centres with limited blood supplies. Consequently, patients are deprived of blood even in life threatening situations [6]. In view of the foregoing and the dwindling health resources, it is important to identify risk factors for blood transfusion in patients undergoing caesarean delivery. This will prevent unnecessary routine cross-matching; ensure judicious and rational use; preserve limited blood supply; thus improve availability without compromising the quality of care.

This study aimed to identify and evaluate risk factors for blood transfusion in women who underwent caesarean delivery at the Obstetric Unit of Lagos State University Teaching Hospital, Ikeja, Nigeria.

## Methods

This is a prospective observational study that was carried out at the obstetric unit of the Lagos State University Teaching Hospital (LASUTH), Nigeria. This hospital is a referral centre for private and public health institutions in Lagos and the neighboring states. Approximately 2000 deliveries take place per annum.

Pregnant women who attended the maternity unit for caesarean deliveries between January 1st and December 31st, 2011 were the participants.

### Definition of terms

A patient was defined as “Transfused” or “Not transfused” when her caesarean delivery was associated or not associated with blood transfusion respectively; at any time prior to discharge. An ‘unbooked patient’ is one who is not registered for antenatal care at LASUTH. Pre-operative anaemia and pre-operative fever were defined as a packed cell volume of less than 30% and temperature of 37.5 °C or more respectively, occurring within 24 h of commencement of surgery. The duration of surgery (skin incision to last stitch) was regarded as ‘prolonged’ when it lasted more than one hour. An estimated blood loss of 1000mls or more at the conclusion of surgery was regarded as excessive. Obesity was defined as body mass index (BMI) equal to or greater than 30 kg/m^2^. The period between 8.a.m. and 4.p.m. on weekdays are referred to as regular hours while call hours refers to all periods outside these hours.

### Data collection

All women who had either elective or emergency caesarean deliveries during the study period consented to participation and were enrolled. Information was obtained directly from patients, their clinical notes and referral letters using structured pre-tested data collection form. Data were recorded on a daily basis by investigators and trained research assistants from admission through delivery till discharge. Women who had vaginal deliveries were excluded.

Socio-demographic data obtained included; age, parity, booking status, maternal weight, height, and educational status. Details of labour; pre-operative morbid conditions such as anaemia, presence of uterine fibroids, previous uterine surgery, chronic hypertension; and gestational age at delivery were amongst the perioperative conditions investigated. Intra-operative and post-operative characteristics obtained included indication for caesarean section, type of anaesthesia (general or regional), type of abdominal incision, cadre of surgeon, surgical events, and duration of surgery. The cadre of surgeon that carried out the caesarean delivery varied according to indication and unit protocol; either consultants or resident doctors. Placenta was delivered by controlled cord traction except where this was difficult in which case manual removal was performed.

Blood loss was estimated by counting the number of soaked abdominal packs and gauzes; measurement of volume of blood expelled from the vagina after caesarean section; and visual estimation of blood staining of the theatre linen and drapes. The intraoperative decision for blood transfusion was made by the attending anaesthesiologist based on preoperative haematocrit, the estimated blood loss and the clinical status of the patient. The attending obstetrician subsequently decided on the postoperative transfusion needs of patients. However, those with massive haemorrhage or consumption coagulopathy are jointly managed with the Haematologists. Though transfusion haematocrit threshold varies, patients in stable clinical condition hardly require transfusion at haematocrit levels of 26% or more. The units of blood transfused were recorded. The aforementioned were all explored as risk factors contributing to blood transfusion in caesarean deliveries.

### Statistical analysis

Information obtained was entered into the computer and analysed with the EPI-Info statistical software 3.5.3 version (2011) of the Centre for Disease Control and prevention (CDC), Atlanta, USA. A comparison between transfused and non-transfused parturients was made using Chi-square, Student’s *t* test and Mann-Whitney-U test where appropriate. A *p* value of <0.05 was considered statistically significant.

Univariable and multivariable logistic regression models were constructed to identify independent risk factors associated with blood transfusion in women with caesarean delivery.

## Results

A total of 2134 deliveries occurred during the study period of which 906 had caesarean section. Thus a caesarean section rate of 42.5% was recorded. Of the 906 that had caesarean section, 188 (20.8%) received blood transfusion. The most common indication for caesarean section was cephalo-pelvic disproportion (25.7%) but only 15.9% of this group had blood transfused, whereas of the 12.2% women who under-went caesarean section for ante-partum haemorrhage 81.51% of the group were transfused.

Other common indications were malpresentation, fetal compromise, hypertensive disorders of pregnancy, repeat caesarean section and the transfusion rate was between 7.1% and 19.4% for these conditions.

**Fig. 1:**
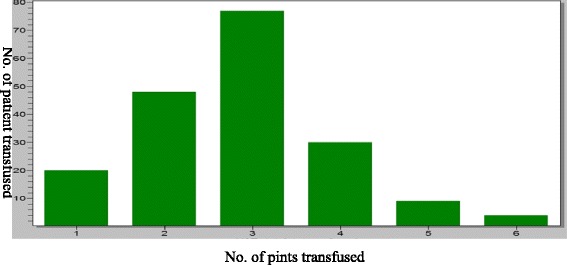
Frequency of units of blood transfused

The mean duration of surgery was 51.1 min (SD = 22.6) with a range of 20–334 min while the pre-operative packed cell volume (PCV) ranged from 20 to 39% with an overall mean of 32.9% (SD = 2.8). The median gestational age of women who had blood transfusion was 39.0 weeks (interquartile range, IR = 38–40) which was same as those who didn’t receive blood transfusion. The highest frequency of blood units transfused was 3, occurring in 41.3% of patients as shown in Fig. 1.

The significant socio-demographic risk factors for transfusion in these women were; age (≥35), social class, parity and obesity with as many as 52.1% of patients who received blood transfusion being of unbooked status (cOR = 3.23, 95% CI = 2.3–4.5) as shown in Table 1.

**Table 1:** Socio-demographic profile of blood transfused versus non-transfused patients

Variables	Transfused (*N* = 188)	Not transfused (*N* = 718)	Crude Odds Ratio(cOR)	95% CI	*P* value
Age group					
< 20	4(50%)	4(50%)	1.00	reference	reference
20–34	158(21.7%)	569(78.3%)	0.28	0.06–1.22	0.07
35 and above	26(15.2%)	145(84.8%)	0.18	0.04–0.76	0.02
Marital status					
Single	37(31.9%)	79(68.1%)	0.94	0.33–2.69	0.90
Married	134(18.8%)	577(81.2%)	0.46	0.17–1.26	0.13
Separated	6(16.2%)	31(83.8%)	0.39	0.10–1.44	0.16
Widowed	5(20.8%)	19(79.2%)	0.52	0.13–2.11	0.37
Divorced	6(33.3%)	12(66.7%)	1.00	reference	reference
Social class					
Upper	20(15.6%)	108(84.4%)	0.48	0.28–0.82	0.01
Middle	67(16.2%)	347(83.8%)	0.50	0.36–0.71	<0.001
Lower	101(27.7%)	263(72.3%)	1.00	Reference	Reference
Parity					
0	49(28%)	126(72%)	1.00	Reference	Reference
1–4	126(19.1%)	532(80.9%)	0.61	0.42–0.89	0.01
5 and above	3(17.8%)	60(82.2%)	0.56	0.28–1.11	0.09
Obesity					
Yes	77(17.5%)	362(82.5%)	0.68	0.49–0.95	0.02
No	111(23.8%)	356(76.2%)	1.00	Reference	Reference
Unbooked Status					
Yes	98(35.1%)	181(64.9%)	3.23	2.32–4.50	<0.001
No	90(14.4%)	537(85.6%)	1.00	Reference	Reference

*Significant *p* value < 0.05

After univariable analysis of all the factors, those found to be significantly associated with risk of blood transfusion at caesarean section were: previous uterine scar irrespective of elective or emergency C/S; cadre of surgeons; prolonged operation time; pre-operative anaemia; general anaesthesia, time of surgery and prolonged labour as depicted in Table 2.

**Table 2:** Obstetrics/Operative factors in blood transfused versus non-transfused patients

Variables	Transfused (*N* = 188)	Not transfused (*N* = 718)	Crude Odds Ratio (cOR)	95% CI	*P* value
Previous uterine scar					
Yes	42(14.1%)	256(85.9%)	0.52	0.36–0.76	<0.001
No	146(24.0%)	462(76.0%)	1.00	Reference	Reference
Elective c/s					
Yes	31(9.9%)	281(90.1%)	0.31	0.20–0.46	<0.001
No	157(26.4%)	437(73.6%)	1.00	Reference	Reference
No. of foetus					
Single	172(21.1%)	643(78.9%)	1.25	0.71–2.21	0.43
Multiple	16(17.6%)	75(82.4%)	1.00	reference	Reference
Cadre of surgeons					
Registrar	58(13.6%)	368(86.4%)	0.04	0.02–0.09	<0.001
Senior registrar	100(22.6%)	343(77.4%)	0.07	0.03–0.16	<0.001
Consultant	30(81.1%)	7(18.9%)	1.00	Reference	Reference
Time of surgery					
Regular hours	87(18.1%)	393(81.9%)	0.07	0.52–0.98	0.04
Call hours	101(23.7%)	87(18.1%)	1.00	Reference	Reference
Preoperative anaemia					
Yes	161(47.1%)	181(52.9%)	17.69	11.38–27.50	<0.001
No	27(4.8%)	537(95.2%)	1.00	Reference	Reference
Anaesthesia					
Regional	102(14.0%)	626(86.0%)	0.17	0.12–0.25	<0.001
General	86(48.3%)	92(51.7%)	1.00	Reference	Reference
Prolonged operation time					
Yes	111(55.5%)	89(44.5%)	10.19	7.07–14.69	<0.001
No	77(10.9%)	629(89.1%)	1.00	Reference	Reference
Prolonged labour					
Yes	38(46.9%)	43(53.1%)	3.46	2.09–5.75	<0.001
No	73(20.3%)	286(79.7%)	1.00	Reference	Reference

*significant *p* = <0.05

Pre-existing conditions such as hypertension, pre- operative fever and uterine fibroids are shown in Table 3, while indications for caesarean section and second stage caesarean section (C/S) data are presented in Table 4.

**Table 3:** Pre-pregnancy morbidities in transfused versus non- transfused patients

Variables	Transfused (*N* = 188)	Not transfused (*N* = 718)	(cOR)	95% CI	*P* value
Chronic Hypertension					
Yes	9(7.2%)	116(92.8%)	0.26	0.13–0.52	<0.001
No	178(23.0%)	596(77.0%)	1.00	Reference	Reference
Diabetes					
Yes	4(23.5%)	13(76.5%)	1.18	0.38–3.66	0.78
No	184(20.7%)	705(79.3%)	1.00	Reference	Reference
Pre-operative fever					
Yes	20(50%)	20(50%)	4.16	2.19–7.90	<0.001
No	168(19.4%)	698(80.6%)	1.00	Reference	Reference
HIV					
Yes	11(26.2%)	31(73.8%)	1.38	0.68–2.79	0.38
No	177(20.5%)	687(79.5%)	1.00	Reference	Reference
Uterine fibroids					
Yes	12(70.6%)	5(29.4%)	9.72	3.38–27.96	<0.001
No	176(19.8%)	713(80.2%)	1.00	Reference	Reference
Preoperative anaemia					
Yes	161(47.1%)	181(52.9%)	17.69	11.38–27.50	<0.001
No	27(4.8%)	537(95.2%)	1.00	Reference	Reference

Data presented as *n* (%), crude odds ratio (cOR) and 95% confidence intervals (CI)*Significant p = <0.05

**Table 4:** Indications for caesarean section in transfused versus non-transfused patients

Variable	Transfused (*N* = 188)	Not transfused (*N* = 718)	Crude Odds Ratio(cOR)	95%CI	*P* value
Repeat C/S					
Yes	22(20.8%)	84(79.2%)	1.0	0.61–1.64	0.99
No	165(20.8%)	628(79.2%)	1.0	Reference	Reference
Cephalopelvic disproportion					
Yes	29(12.9%)	195(87.1%)	0.49	0.32–0.75	<0.001
No	158(23.4%)	517(76.6%)	1.0	Reference	Reference
Preeclampsia/Eclampsia					
Yes	64(30%)	149(70.0%)	0.26	1.38–2.80	<0.001
No	124(17.9%)	569(82.1%)	1.0	Reference	Reference
Placenta Previa					
Yes	51(81.0%)	12(19.0%)	21.88	11.36–42.11	<0.001
No	136(16.3%)	700(83.7%)	1.0	Reference	Reference
Abruptio Placenta					
Yes	46(82.1%)	10(17.9%)	22.94	11.31–46.52	<0.001
No	142(16.7%)	708(83.3%)	1.0	Reference	Reference
Non reassuring fetal status					
Yes	17(11.2%)	135(88.8%)	0.43	0.25–0.73	0.002
No	171(22.7%)	583(77.3%)	1.0	Reference	Reference
Malpresentation					
Yes	11(6.9%)	148(93.1%)	0.24	0.13–0.45	<0.001
No	177(23.7%)	570(76.3%)	1.0	Reference	Reference
Second stage C/S					
Yes	30(90.5%)	3(9.1%)	45.25	13.64–150.13	<0.001
No	158(18.1%)	715(71.9%)	1.0	Reference	Reference

Preoperative anaemia was a significant risk factor for blood transfusion (OR = 17.69). The mean packed cell volumes of transfused and non- transfused patients were 29.6% and 33.7% respectively and the difference was statistically significant.

Regional anaesthesia was a protective factor for blood transfusion (OR = 0.17, 95% CI 0.12–0.25) while co-existing uterine fibroid increased the odds of transfusion. (OR = 9.72, 95% CI 3.38–27.96).

Multiple logistic regression analysis of the significant factors was performed and independent risk factors were determined.

Second stage C/S (aOR = 76.14, 95% CI = 1.25–4622.06, *p* = 0.04), placenta previa (aOR = 32.57, 95% CI = 2.22–476.26, *p* = 0.01), placental abruption (aOR = 25.35, 95% CI = 3.06–211.02, *p* < 0.001), pre-operative anaemia (aOR = 12.15, 95% CI = 4.02–36.71, p < 0.001), prolonged operation time (OR = 10.72 95% CI = 1.37–36.02, p < 0.001), previous uterine scar (aOR = 7.02, 95% CI = 1.37–36.02, *p* = 0.02) and hypertensive disorders in pregnancy (aOR = 5.19, 95% CI = 1.84–14.68, p < 0.001) were found to be significantly higher risk factors for blood transfusion at caesarean section while obesity was found to be a protective risk factor (aOR = 0.24, 95% CI = 0.09–0.61, *p* = 0.002) as depicted in Table 5.

**Table 5:** Risk factors for caesarean-related blood transfusion: Multivariate analysis

Independent risk factors	aOR	95% CI	*P*-value
Age group (35 years or more versus <20 years)	0.02	0.00–1.79	0.09
Age group (20–34 versus <20 years)	0.02	0.00–1.67	0.09
Obesity (Yes/No)	0.24	0.10–0.61	<0.001
Pre-op anaemia (Yes/No)	12.15	4.02–36.71	<0.001
Anaesthesia (Regional/General)	0.37	0.13–1.07	0.07
Booked (Yes/No)	1.99	0.76–5.23	0.16
CPD (Yes/No)	0.57	0.08–3.93	0.57
Chronic Hypertension	0.60	0.07–5.30	0.64
Placenta previa (Yes/No)	32.57	2.23–476.26	0.01
Abruptio placentae (Yes/No)	25.35	3.05–211.02	<0.001
Fetal conditions (Yes/No)	1.13	0.13–9.47	0.91
Mal-presentation (Yes/No)	0.88	0.09–8.28	0.10
Prolonged labour (Yes/No)	1.90	0.62–5.87	0.26
2ndstageCs (Yes/No)	76.13	1.25–4622.06	0.04
Pre eclampsia/eclampsia (Yes/No)	5.19	1.84–14.68	<0.001
Pre-0pfever (Yes/No)	3.14	0.76–13.06	0.12
Fibroids (Yes/No)	1.31	0.11–15.75	0.84
Previous uterine scar (Yes/No)	7.02	1.37–36.02	0.02
Elective C/S (Yes/No)	0.03	0.00–46.39	0.34
Prolonged operation time (Yes/No)	10.72	4.20–27.33	<0.001
Cadre of surgeon (Registrar/Consultant)	5.32	0.10–285.63	0.41
Cadre of surgeon (Senior Registrar/Consultant)	1.21	0.02–70.36	0.93
Time of surgery (Regular hours/Call hours)	1.05	0.41–2.69	0.92
Parity grouped (1–4/0)	0.39	0.14–1.07	0.07
Parity grouped (5 and above/0)	0.30	0.03–2.66	0.28
Social class (middle/lower)	0.85	0.31–2.32	0.75
Social class (upper/lower)	0.52	0.13–2.09	0.36
CONSTANT	reference	reference	0.78

Adjusted odds ratio (aOR) and 95% confidence intervals (CI) *Significant p = <0.05

## Discussion

In this prospective study of 906 women undergoing caesarean delivery, we found an overall risk of caesarean-associated blood transfusion of 20.8%, which is high compared with 0.63% to 12.21% found in similar studies done in Australia, the United States of America, Denmark and India [3, 4, 7–9]. The high transfusion rate in this study may be explained by the high incidence of emergent cases such as prolonged labour, cephalo-pelvic disproportion and the relatively large number of cases of placenta previa, abruptio placentae and hypertensive disorders in pregnancy (pre-eclampsia/eclampsia).

In a study of blood transfusion in obstetric practice in Lagos University Teaching Hospital (LUTH), Lagos, a sister tertiary centre, the overall transfusion rate was 12.1% [6] lower than the 20.8% in this study. In an earlier study done in our centre by Akinola et al. [10], the blood transfusion rate was 12.5% which is significantly lower than the transfusion rate in this study. This lower figure may be explained by the shorter duration of that study (3 versus 12 month) and the potential underestimation and errors from incomplete data entries inherent in retrospective studies.

The caesarean section rate in this study was 42.5% which is high compared to the total U.S. caesarean delivery rate of 32.9% of all births in 2009 [11] and 5–21.8% reported in Sub-Saharan Africa [12]. The World Health Organization however recommends a caesarean section rate of 5–15% in any facility [13]. High caesarean delivery rates have been an issue of international concern, though most cases in this study were emergency surgeries with genuine indications. Our facility also serves as a major tertiary referral centre for Lagos metropolis with a population of about 18 million inhabitants which translates to a very large patient load.

The practice of routine cross matching of blood for women undergoing caesarean section in our centre, in anticipation of significant haemorrhage, irrespective of indication, may inadvertently result in unnecessary transfusions as the anaesthetist or obstetrician who might not have recommended a transfusion does so because the blood is available in the theatre.

In this study, second stage Caesarean delivery was an independent risk factor for blood transfusion (aOR = 76.14, 95% CI = 1.25–4622.06, *p* = 0.04). The risk of blood transfusion at second stage section was well stated in the study by Allen et al. that the maternal risks of second stage caesareans included major haemorrhage, greater risk of bladder trauma, and extension tears of the uterine angle leading to broad ligament haematoma [14]. Other studies have similarly reported increased frequency of uterine atony; the need for hysterectomy is also found to be more frequent in the Caesarean deliveries performed in the second stage of labour. This atony has been attributed to longer labour duration resulting in uterine hypotonia. The increased frequency may suggest that these operations are technically more difficult [14, 15]. In advanced cephalo-pelvic disproportion, when the head has entered deep into the pelvis, manipulating the trapped head may lead to lateral extension of the uterine incision with attendant massive haemorrhage [16]. Uterine atony and other causes of haemorrhage resulted in a significantly higher transfusion requirement in women undergoing Caesarean deliveries in the second stage of labour [17]. The Royal College of Obstetricians and Gynaecologists in the UK, suggests that a consultant be present at all second stage Caesarean deliveries to make an informed decision and to reduce complications arising from such operations [18].

Our antepartum haemorrhage indication for caesarean delivery of 12% is higher than 8–10% reported by a Medicins sans Frontiers multi-country analysis conducted in sub-Saharan Africa [19]. Incomplete evaluation for the aetiology of antepartum haemorrhage in unbooked patients coupled with obstetricians’ reluctance to allow vaginal delivery even in cases of minor placental previa may be contributory. Placenta previa and Abruptio placentae are the major causes of ante-partum haemorrhage. This study shows that majority of the blood transfusions were indicated for antepartum haemorrhage (placenta previa and abruption) and much fewer transfusions for other indications. Of the 63 women with placenta previa, 51 were transfused (aOR = 32.57, 95% CI = 2.22–476.26, *p* = 0.01) and of the 56 women with abruptio placentae, 46 were transfused (aOR = 25.35, 95% CI = 3.06–211.02, *p* < 0.001). This shows a very significant risk for blood transfusion.

Pregnancies complicated by placenta previa are noted for increased blood loss and transfusion at surgery. Factors responsible include repeated ante-partum haemorrhage which may lower the haematocrit, thus putting the patient at a point close to transfusion threshold. Similarly, the low-lying placenta may provoke increased and uncontrollable intra-operative haemorrhage necessitating blood transfusion [5]. Abruptio placentae may be complicated by disseminated intravascular coagulopathy (DIC), couvelaire uterus, uterine atony and uterine rupture. These would further increase the risk of blood transfusion although these complications were not ascertained in the cases of ante-partum haemorrhage in this study.

In our centre, the most senior obstetrician conducts the delivery in cases of ante-partum haemorrhage as there might be recourse to hysterectomy. Research also suggests careful perioperative planning whenever placenta previa complicates a previous caesarean delivery. It can be inferred from this study that all patients with placenta previa or abruptio placentae should have blood cross matched for caesarean delivery.

Pre-operative anaemia was an independent risk factor for blood transfusion (aOR = 12.15, 95% CI = 4.02–36.71, *p* < 0.001). This was not surprising as a woman with anaemia will tolerate less any amount of blood loss and may develop cardiovascular compromise from haemodynamic instability. Antenatal care has been shown to positively influence haematocrit value in a population of Nigerian women [20]. Such care would identify complications of pregnancy and enable a goal-directed approach to labour and delivery. These risks also argue for optimizing maternal antenatal iron status to avoid severe anaemia and they suggest that informing severely anaemic iron deficient women about their high risk of transfusion should they undergo caesarean section might enhance compliance with iron supplementation. Thus, the panacea appears to be improved antenatal care. Meanwhile, patients who are presenting in labour without adequate prenatal care to mitigate anaemia should have donor blood cross-matched for caesarean delivery. Antenatal and preoperative anaemia should be corrected vigorously. Pregnant women should not approach term or go into spontaneous labour while still anaemic [20]. The role of oral iron supplements in correcting anaemia is well-documented [21].

In this study, multivariable analysis using the logistic regression model showed previous uterine scar as a risk factor for blood transfusion (aOR = 7.02, 95% CI = 1.37–36.02, *p* = 0.02) in contrast to the initial univariable analysis which suggested a reduced blood transfusion risk. (cOR = 0.52, 95% CI = 0.36–0.76, *P* = 0.001). The higher rate of transfusion in patients without uterine scar compared with those with scar (24.0% versus 14.1%) might be due to cases of abruptio placenta, preoperative anaemia and second stage sections occurring in those without uterine scars. The increased blood transfusion risk may be explained by the higher incidence of intra-operative complications such as adhesions, extension of uterine incision, atony and the need for hysterectomy. All these may be responsible for prolonged operation time (aOR = 10.72 95% CI = 1.37–36.02, *p* < 0.001) when compared to those without complications.

Obesity was found to reduce the risk for blood transfusion at caesarean section (aOR = 0.24, 95% CI = 0.09–0.61, *p* = 0.002). This finding appears surprising as one would expect more challenges at surgery and also the possibility of prolonged operation. However the reduced risk may be as a result of more experienced surgeon operating these patients because of the anticipated problem.

With an increasing incidence of abdominal delivery, the risk of requiring blood transfusion is still significant especially in high risk cases [22]. Improvements in obstetrics surgical techniques and practice, physicians’ acceptance of lower peri-operative haemoglobin concentration and adoption of more restrictive indications for blood transfusion, will no doubt contribute to a decrease in blood transfusion rate. Additionally, the challenge posed by patients’ reluctance to receive homologous blood transfusion because of the risk of transmission of blood borne infectious agents and the fact that the obstetrics population is largely young and healthy [23] will by necessity compel obstetricians and anaesthetists [24] to consider a trend towards minimal transfusion.

### Strengths and limitation

The strength of this study is the prospective nature of its design. Although this study was performed at a single institution, the data may be of a regional significance due to the institution’s unique geographic location and patient volume. A larger sample size might have provided additional power to show statistically significant differences for some of the risk factors. Though a number of the factors appear to be statistically significant, caution has to be exercised in their interpretation because of the wide confidence intervals.

## Conclusion

The overall risk of blood transfusion in cesarean delivery is high. Parturients with second stage Caesarean section, placenta previa, abruptio placentae and preoperative maternal anaemia have an increased risk of blood transfusion. Careful evaluation of patients for such risks factors prior to surgery coupled with adequate peri-operative preparations for blood transfusion would be expected to ensure optimal blood utilization and better maternal outcome. More so in settings with limited blood supplies and where haemorrhage plays a greater role in maternal mortality. Optimizing maternal hemoglobin concentration during antenatal period may reduce the incidence of caesarean-associated blood transfusion.

## Abbreviations

CDC: Centre for Disease Control and prevention
LASUTH: Lagos State University Teaching Hospital
LUTH: Lagos University Teaching Hospital, Lagos
